# Reduced Social Risk-Taking in Depression

**DOI:** 10.1037/abn0000797

**Published:** 2023-02

**Authors:** Daisy Follett, Caitlin Hitchcock, Tim Dalgleish, Jason Stretton

**Affiliations:** 1 Medical Research Council Cognition and Brain Sciences Unit, University of Cambridge; 2 Cambridgeshire and Peterborough NHS Foundation Trust, Cambridge, UK

**Keywords:** depression, risk taking, social psychology, evolutionary theory

## Abstract

Evolutionary models of depression posit that depressed mood represents an adaptive response to unacceptably low social status, motivating the inhibition of social risk-taking in favor of submissive behaviors which reduce the likelihood of social exclusion. We tested the hypothesis of reduced social risk taking using a novel adaptation of the Balloon Analogue Risk Task (BART) in participants with major depressive disorder (MDD; *n* = 27) and never-depressed comparison participants (*n* = 35). The BART requires participants to pump up virtual balloons. The more the balloon is pumped up, the more money a participant gains on that trial. However, more pumps also increase the risk the balloon will burst such that all money is lost. Prior to performing the BART, participants took part in a team induction in small groups in order to prime social-group membership. Participants then completed two conditions of the BART: an Individual condition where they risked only their own money, and a Social condition, where they risked the money of their social group. The groups did not differ in their performance in the individual condition (Cohen's *d* = 0.07). However, the MDD group risked fewer pumps in the Social condition than the never-depressed group (*d* = 0.57). The study supports the notion of an aversion to social risk-taking in depression.

## General Scientific Summary

Evolutionary models of depression suggest that depressed mood evolved to protect low-ranking individuals from social exclusion. This study found that depressed individuals are more careful with group resources than healthy participants, or than with personal resources, providing evidence consistent with reduced social risk-taking in depression.

Depression is one of the most prevalent health conditions in the world, affecting more than 300 million people, and is the third leading cause of years lived with disease worldwide (Global Burden of Disease Study, 2018). It is linked with disruption to education, marital status, and employment (R. C. Kessler, 2012), and research shows that the detriment to perceived health associated with depression is greater than that associated with cancer, cardiovascular disorders (Alonso et al., 2011), arthritis, or diabetes (Moussavi et al., 2007).

The Social Risk Hypothesis (SRH) of depressed mood (Allen & Badcock, 2003) posits that depressed states evolved during the human era of evolutionary adaptation in part to protect the individual's membership in a social group. A defining tenet of the SRH is thus the adaptive inhibition of social risk-taking behaviors in individuals who perceive their social status as critically low such that adverse social outcomes could result in expulsion from the social group. Social risk-taking constitutes action(s) or behavior(s) which have possible negative outcomes for the social status of the individual; for instance, challenging another group member for a valued resource, making a decision that confers danger for the group, acting selfishly. According to SRH, inhibition of social risk-taking is most adaptive when the individual possesses a critically low “social investment potential.” That is, when the ratio between the individual's self-perceived social value to the group relative to their social burden is weighted such that the perceived burden exceeds the perceived value. In these circumstances, the perceived probability of exclusion from the social group should a socially risky choice or behavior backfire is deemed to be high. However, such change in behavior is only functional to the extent that perceptions of social value and social burden are valid. When depressed mood becomes maladaptive, for example in those with clinical depression, the proposal is that these cognitive evaluations of value and burden can become biased or distorted (Kupferberg et al., 2016), with consequent implications for social behavior.

Although risk-taking in a broad sense has been well investigated in clinically depressed samples, social risk-taking specifically has received relatively little attention, despite evidence of the importance of domain specificity in risk-taking preferences (Bran & Vaidis, 2020; Hanoch et al., 2006; Weber et al., 2002). Socio-economic games provide an opportunity to investigate social risk-taking experimentally; these approaches comprise a range of experimental paradigms which combine methodologies from social-cognitive science and economics to investigate the psychological underpinnings of economic and social decision-making. These paradigms allow social decision-making to be evoked and measured under controlled laboratory settings and thus provide an appropriate methodology for studying maladaptive processes in psychiatric conditions (King-Casas & Chiu, 2012). The purpose of this study was therefore to investigate the specific interaction between social risk-taking and clinical depression guided by the predictions of the SRH.

Self-report (as opposed to experimental) measures of risk-taking have indicated increased reported risk-taking in depression, with a positive relationship between overall risk-taking and depression-relevant constructs such as anhedonia (Pushkarskaya et al., 2019), as well as a positive relationship between depression and recreational risk-taking (Khodarahimi & Fathi, 2016). With respect to social risk, depressive symptomology is positively related to self-reported heightened levels of concern for such risks (Andrews et al., 2020). Studies surveying real-world behavior using metrics such as gambling or dangerous driving habits to quantify “riskiness,” also indicate a greater propensity for risk-taking in depression. Depressive symptoms have been associated with risky behaviors such as non-seat-belt use, engaging in physical fights, or riding with a drunk driver (Pesa et al., 1997), risky sexual behavior (Coyle et al., 2019; Kosunen et al., 2003; Sales et al., 2011) and problematic gambling (Takamatsu et al., 2016).

Overall, despite some inconsistency, there is a general trend toward increased risk-taking in depression. However, the known confounds associated with self-report measures alongside the difficulty of controlling for relevant societal and ecological factors in real-world research such as driving behavior makes it difficult to draw robust conclusions about the underlying constructs. Given these limitations and in the light of the specific predictions of the SRH that social risk-taking will be reduced in depression, the present study used an experimental task in the laboratory to investigate social risk-taking specifically.

The task was a socially adapted version of the well-established behavioral assessment of risk taking, the BART (C. W. Lejuez et al., 2002). In the BART, participants press a response key to inflate a computerized balloon. Reward tokens are accrued in proportion to the size of the balloon, so there is an incentive to inflate the balloon to be as large as possible through repeated presses, or “pumps.” However, the risk exponentially increases as the balloon is inflated, as the balloon may burst at any time, thus losing all of the accrued reward tokens. Participants may save (or “bank”) their tokens at any time; however, this returns the balloon to its minimum size. Evaluations of the BART have found it has high validity in predicting real-world risk-taking behavior; for instance, correlating with drug abuse and alcohol usage (Aklin et al., 2005), gambling and smoking (C. Lejuez et al., 2003; C. W. Lejuez et al., 2002), in addition to trait measures of risk-taking and impulsivity (Hunt et al., 2005). In those with depression, performance on the BART is associated with reduced risk-taking and increased sensitivity to punishment (Hevey et al., 2017). Relative to never-depressed peers, depressed participants made significantly fewer pumps of the balloon overall and reduced the number of pumps after loss trials (when the balloon burst). Moreover, all participants were informed, prior to the task beginning, of the optimum number of pumps for trial success yet the depressed group pumped the balloon significantly below the known optimum level. However, participants were playing only for their own gain, and did not have to risk the resources of the other participants. This therefore limits the interpretation of risk aversion to an individualistic level (Hevey et al., 2017).

In the current study, a novel modification was made to the BART to allow investigation of the social dimension of risk-taking that is critical to the SRH. Participants performed the BART once for themselves, and once for their “team,” an established social group of fellow participants who were present throughout the task (although they were unable to directly observe the player's screen). In terms of the SRH, the degree to which a participant inflates the balloon to accrue more tokens when playing with the team's money would be an index of “social risk”—it could produce greater payoffs for the team, but with a greater risk of losing the team's money with consequent adverse implications for the individual's status within the group. The degree of such social risk can be mitigated by cautious play when gambling with the team's resources, settling for smaller wins and banking smaller sums to avoid potential losses. Examining individual player strategies alongside their clinical status therefore provides an opportunity to examine our prediction that depressed mood will result in the inhibition of specifically socially risky behavior, relative to a control condition when participants are playing the BART with their personal funds only.

## Hypotheses and Aims

In line with the predictions of the SRH, our hypothesis was that depressed individuals would exhibit greater risk aversion/reduced risk-taking in the social condition of the BART (expressed as a lower number of balloon pumps per trial) relative to never-depressed controls, and compared to the individual condition.

## Method

### Participants

Power analysis (G*Power 3.1, Faul et al., 2007) estimated that 26 participants were required in each group for a two-tailed α of .05, to provide 90% power to detect a moderate effect size of Cohen's *f* = 0.2, assuming a correlation between the two BART conditions of 0.6, for the interaction between Condition and Group. We therefore sought to recruit at least 30 participants in each group to allow for any attrition. Our sample comprised 65 adults (41 female, range 18–64, mean = 44 years) recruited from the MRC CBU volunteer panel. They included 35 control participants (23 female, age range 18–64, mean = 42 years) and 30 clinically depressed participants (18 female, age range 21–64, mean = 46 years). For both groups, inclusion criteria were normal or corrected-to-normal vision, being over 18 years, and English speaking. Exclusion criteria for both groups were self-reported current experience of psychosis, alcohol, or drug use disorder, a major neurological condition, a diagnosed specific learning disability or the presence of metal in the body (as there was a corollary neuroimaging study). Inclusion criteria for the depressed group were a diagnosis of MDD with a current major depressive episode, according to the fifth edition of the Diagnostic and Statistical Manual of Mental Disorders (DSM-5) as determined by the Structured Clinical Interview for the DSM (SCID; First, 2015). SCIDs were administered by a clinical psychologist (CH) approximately 1 month prior to the study days. Exclusion criteria for the Control group were any current or past mood, anxiety or stressor-related psychiatric disorder according to the DSM-5.

Recruitment took place in Cambridge, the United Kingdom. Individuals who had previously indicated an interest in taking part in psychological studies were emailed and invited to a telephone screening session. As an initial screen, to ensure depressed participants were still in episode, individuals were administered the Mood Module of the SCID via telephone 1 week prior to the testing session (performed by DF^1^). In all cases patients had remained in the episode. Eligible participants were then invited to take part. Matching of the control sample was done at the group level, on the basis of age, gender and educational background. Due to the risk of drop-out in clinical populations, our protocol for group-testing included seeking to book back-up participants for all testing sessions to be utilized where needed. However, no-back up participants were used for the groups reported here all of which had a full complement of five participants. All participants provided written informed consent and were debriefed following completion of the tasks. The study was approved by the Cambridgeshire Research Ethics Committee.

### Protocol

In order for participants to develop social allegiance to a team, the BART was administered within a larger protocol that involved performing a number of tasks as an interacting group (online supplemental materials A) on the same day. Participants were then scheduled for a second individual testing session the day after. Participants therefore did not see each other again after the end of the first day of testing. Importantly, immediately prior to performing the BART task, participants collaborated on a Public Goods Game (PGG) with the four other participants who were present in the same room throughout. The PGG involved making contributions of monetary tokens to a group pot and gave the option to punish those who did not contribute their fair share. This task was chosen due to its emphasis on social responsibility; the group receives the highest gains when every player contributes the maximum possible, relying on each other to do the same, and there is a possibility for those who shirk their social responsibility to be sanctioned. This task therefore seems well placed to impart a sense of collaboration, social responsibility, and allegiance within an experimental setting, with the purpose of increasing the ecological validity of the social component of the current task. The groups were clinically homogenous, comprising either exclusively Depressed or Control participants. Participants were not explicitly informed of this but were told they had been grouped with people who saw the world similarly to themselves. One week prior to these tasks, participants completed a battery of questionnaires related to mood and social rank to characterize and validate differences between groups, detailed below.

### The Social BART

**Figure 1 fig1:**
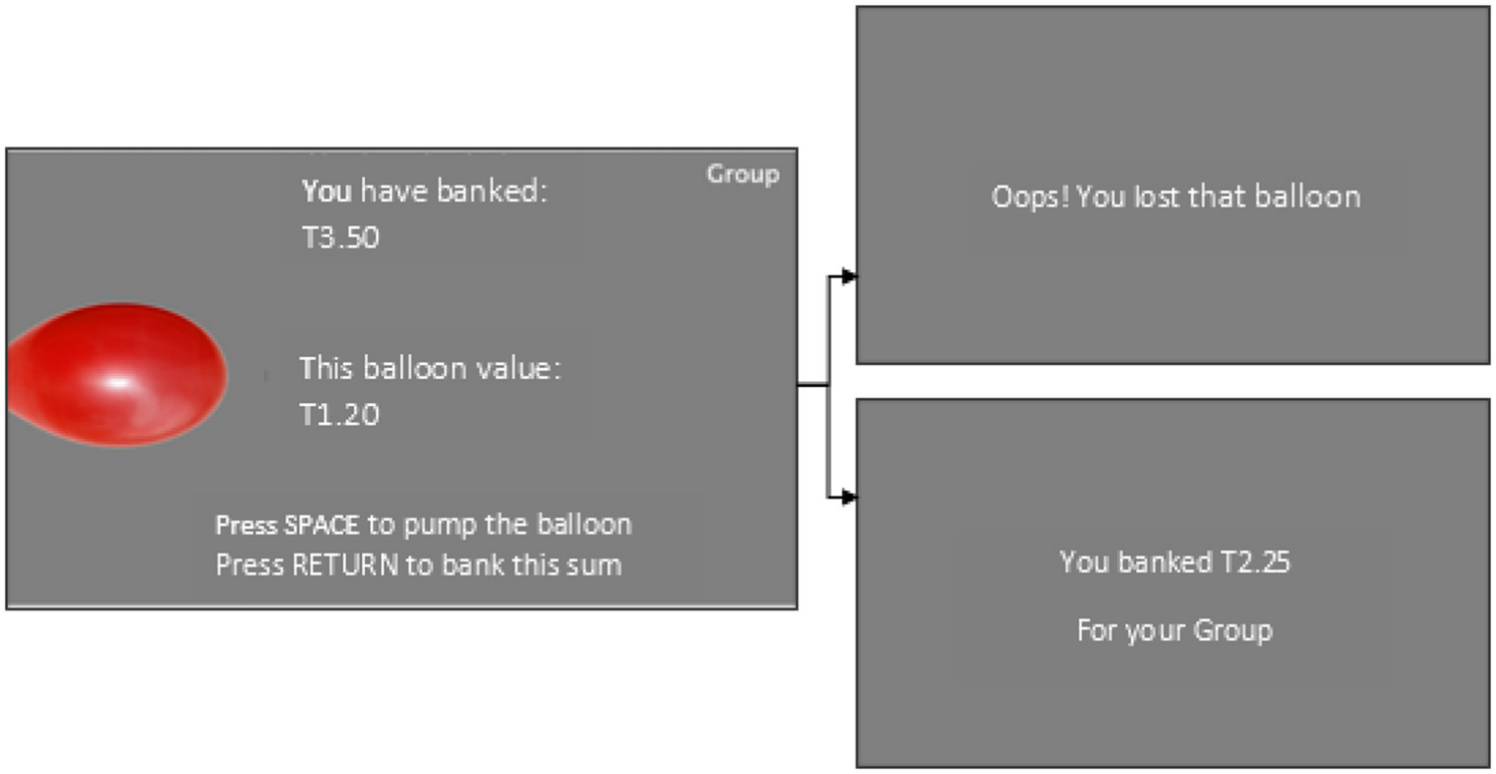
The BART as it was Presented to Participants, Including “Loss” and “Bank” Screens *Note*. See the online article for the color version of this figure.

Participants were seated in a communal testing room with four fellow participants and the experimenter. The BART results were not shared with the group in real-time and each group member was unaware of how much the other group members had “banked” under each condition. This differentiated the social-risk measure from the prior PGG in which participants were able to see how the other group members performed, allowing us to investigate social risk and responsibility under pseudo-anonymous conditions to complement the larger protocol. The Social BART was administered on individual testing laptops, which were positioned such that the screen was visible only to the individual participant. The task was programmed using PsychoPy, a specialized software for experimental psychology, and the visual layout based on Peirce (2019). Prior to the task, participants saw an on-screen information page (online supplemental materials B) detailing the task procedure and monetary value of the tokens. Crucially, participants were informed that they would play two versions of the task, “Individual” and “Social”; in the Individual condition they were playing only for themselves, whereas in the Social condition they were playing for the group and would share their wins with the other group members. Participants were instructed not to speak with one another during the task and not to disclose their totals to each other after the task had finished, and this was monitored for compliance by the experimenters. However, participants were informed that the total scores would be publicized to the rest of the group (though not the individual contributions) the following day in the second testing session and form part of the overall compensation for taking part. The information screen was read aloud by the experimenter, and there was an opportunity to ask questions (Figure 1).[Fig fig1]

Participants played the game once in the individual condition and once in the social condition, with the order counterbalanced across participants. After the initial information screen, participants saw a second screen informing them of which condition they were playing, and an indicator of the condition remained on-screen throughout the task. Participants pressed the space key to inflate, or “pump,” a red balloon presented on the screen, to accrue tokens, with bigger balloons accruing more tokens. When they wished to save, or “bank,” their tokens for that balloon they pressed the enter key, and the balloon would then return to its minimum size. Instructions for the “pump” and “bank” keys remained on-screen throughout. Also on-screen was a running total for the current balloon, as well as a total for the task. The balloons were programed to burst at between 1 and 120 pumps, with an average breakpoint of 60. This was achieved using the same method as C. W. Lejuez et al. (2002), in which an array of numbers 1–120 was constructed, and a number chosen from the array in each round, with the balloon bursting if the number 1 was selected. As numbers were not replaced into the array after selection, the risk of bursting increased with each pump. If the balloon burst before a player had banked their tokens they were presented with a screen which informed them that the tokens had been lost. The dependent variable was adjusted average pumps, representing the average number of pumps across all balloons in that condition which did not burst (C. W. Lejuez et al., 2002) and this was calculated at the individual level for both the Individual and Social conditions. The traditional BART shows high test–retest reliability (White et al., 2008; Xu et al., 2013)

### Questionnaires

All self-report questionnaire measures were collected in the week immediately prior to testing. To characterize differences between experimental groups, two measures of symptomatology and four measures of social status/interaction processing were administered. The Beck Depression Inventory-II (BDI-II; Beck et al., 1996) and Beck Anxiety Inventory (BAI; Beck et al., 1988) were included as measures of symptomatology. The BDI-II is a 21-item self-report inventory, widely used for assessing severity of depression in adolescent and adult populations. Although the BDI-II is not a diagnostic tool, it has been found to have high validity in discriminating between depressed and non-depressed subjects, in addition to high test–retest reliability and construct validity (Richter et al., 1998). The BAI is a 21-item self-report inventory for assessing the severity of anxiety in psychiatric populations. The BAI has been shown to have high internal consistency and retest reliability and is significantly less confounded by depression than other measures of anxiety (Fydrich et al., 1992).

Three measures relevant to social status processing were included to characterize differences in areas of social behavior between groups. The Submissive Behavior Scale (SBS; Allan & Gilbert, 1997) is a 16-item measure, adapted from Buss and Craik (1986), which assesses the type of submissive social behavior that the SRH posits is associated with depressed status. The Involuntary Subordination Questionnaire (ISQ; Sturman, 2011) is a 32-item inventory which measures feelings of inferiority, entrapment, defeat and submissive self-perceptions. The Striving to Avoid Inferiority Scale (SAIS; Gilbert et al., 2007) is a 31-item self-report inventory which measures two factors relating to inferiority; insecure striving—striving based on fear of rejection or criticism—and secure non-striving—a feeling of being socially acceptable regardless of success or failure. Gilbert et al. (2007) found that both factors were significantly related to self-reported fears of rejection, need for validation and feelings of inferiority, and significantly predicted mental health difficulties. The Interpersonal Sensitivity Measure (IPSM; Boyce and Parker, 1989) is a 36-item questionnaire that measures interpersonal hypersensitivity to social interactions, which has been proposed as a possible premorbid personality for depression.

### Transparency and Openness

All data presented in this article are publicly available via the MRC Cognition and Brain Sciences Unit Data Repository (https://www.mrc-cbu.cam.ac.uk/publications/opendata/). This study was not preregistered.

## Results

Three participants from the Depressed Group were excluded from analysis due to missing data giving a final sample size of 62. This still exceeded our a priori power calculation of *n* = 26 per group.^2^ Demographics of the final participant sample are summarized in Table 1. There were no significant differences between groups in relation to gender, age, ethnicity, or education level. Comorbidities are common among presentations of mental health disorders (R. Kessler et al., 2005), therefore, in line with existing literature, participants with common comorbidities were not excluded (Table 2).[Table tbl1][Table tbl2]

Independent samples *t*-tests, as expected, found significant differences between Depressed and Control groups on all three measures of social behavior and both measures of symptomatology (Table 3) in the expected directions.[Table tbl3]

### Social BART Performance

**Figure 2 fig2:**
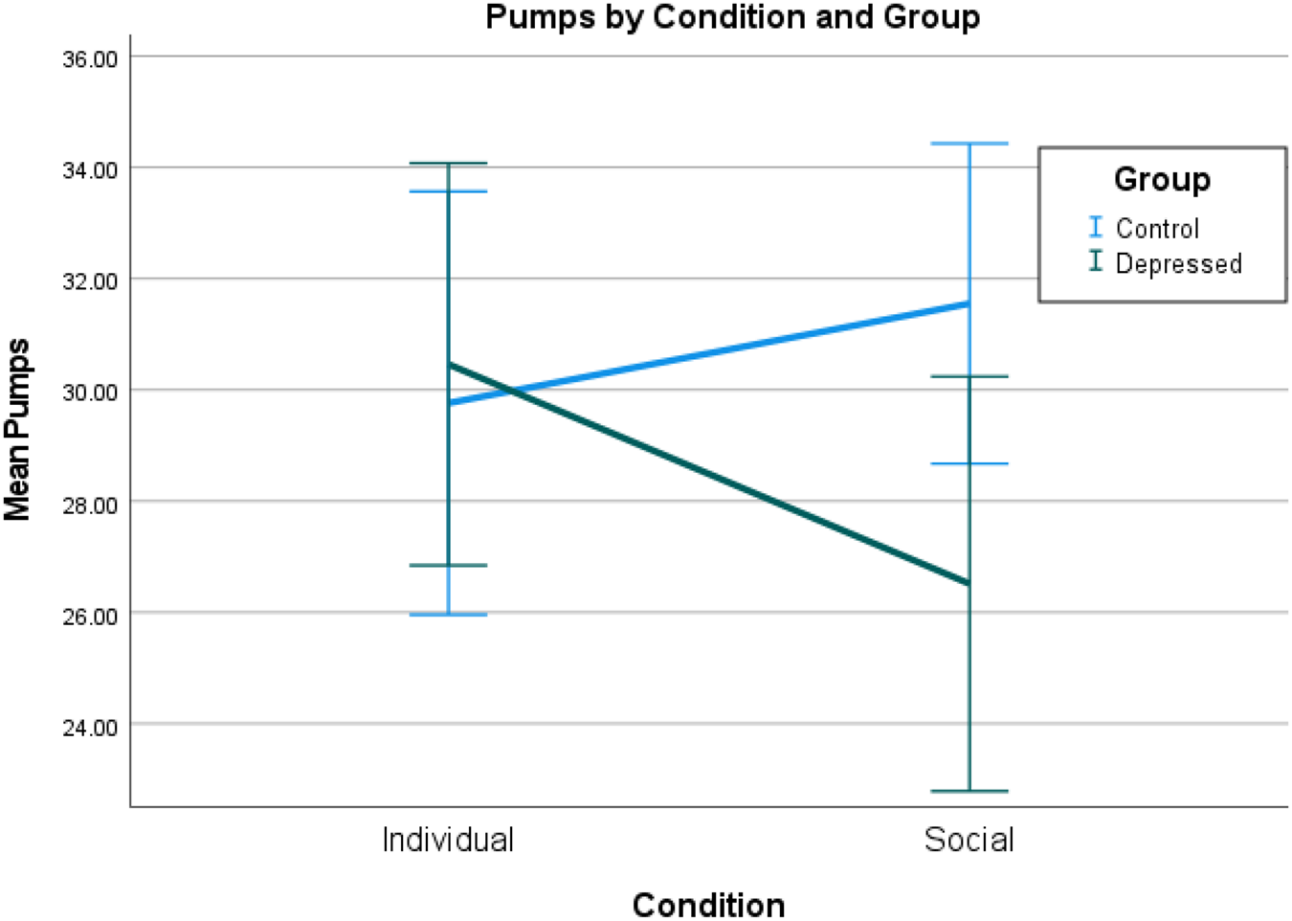
Performance of the Depressed and Control Groups on the Social and Individual Conditions of the Social Balloon Analogue Risk Task *Note*. Error bars = 95% confidence intervals. See the online article for the color version of this figure.

The data for the two groups on the Social BART are presented in Figure 2. A two-way repeated-measures ANOVA with the adjusted mean number of pumps per trial as the dependent variable, Condition (Individual, Social) as the within-subjects factor, and Group (Depressed, Control) as the between-subjects factor, found no significant main effect of Condition, *F*(1, 60) = 1.45, *p* = .23, η*_p_*^2^ = 0.02. Consistent with our hypothesis, there was however a significant interaction of Group by Condition, *F*(1, 60) = 10.24, *p* = .001, η*_p_*^2^ = 0.15.[Fig fig2]

Breaking this interaction down, simple main effects revealed that the Depressed group made significantly fewer pumps in the Social condition compared with the Individual condition, *F*(1, 60) = 8.59, *p* = .005, η*_p_*^2^ = 0.13, whereas within the Control group, there was no significant difference between conditions, *F*(1, 60) = 2.29, *p* = .14, η*_p_*^2^ = 0.04. Furthermore, the depressed group made significantly fewer pumps during the Social condition than did Controls, *F*(1,60) = 4.94, *p* = .03, η*_p_*^2^ = 0.08, but that there was no significant difference between groups during the individual condition, *F*(1, 6) = 0.07, *p* = .79, η*_p_*^2^ = 0.01.

To investigate alternative interpretations of the data, four further analyses were conducted. To investigate whether the seven individual difference measures (BDI, BAI, IPSM, ISQ, SAIS-I, SAIS-II, and SBS) might explain our observed results, Pearson correlations were conducted between these and the adjusted mean number of pumps per trial. There were no significant correlations between any measure and BART performance on either the Individual or Social conditions (all *p*s > .05) suggesting these individual differences did not influence BART performance (see online supplemental materials C). Reinforcing this, an additional repeated-measures ANCOVA including all measures as covariates revealed the critical Group (Depressed, Control) by Condition (Individual, Social) interaction remained significant, *F*(1, 49) = 9.79, *p* = .03, η*_p_*^2^ = 0.17. Given the relevance of Anxiety to risk-taking preferences (Giorgetta et al., 2012) and its high comorbidity with Depression (Kalin, 2020) the potential moderating effects of this variable, as measured by the BAI, were investigated further utilizing a repeated measures ANCOVA (online supplemental materials D). This found no significant interaction between BAI score and the difference in number of pumps between the two conditions, *F*(1, 60) = 0.05, *p* = .82, η*_p_*^2^ = 0.00, and the aforementioned critical interaction reported between Condition and Group remained significant, *F*(1, 60) = 6.77, *p* = .012, η*_p_*^2^ = 0.11. Finally, to investigate the potential impact of the previously completed PGG on performance of the Social condition of the BART, independent samples *t*-tests were conducted comparing PGG performance between the two BART-task groups and, at the individual level, Pearson correlations were conducted between PGG performance and subsequent adjusted average pumps on the BART. There were no significant group differences on any measure of the PGG. There were also no significant correlations observed between the number of pumps an individual made in the Social Condition of the BART and either the Total Tokens accrued or the number of Punish points allocated by their group during the PGG (all *p*s > .05) indicating that performance in the PGG did not significantly influence socially relevant performance in the BART task (see Supplemental Materials E).

## Discussion

This study aimed to evaluate the extent to which currently depressed individuals show a specific reduction in social risk-taking relative to personal risk taking and relative to never-depressed peers, measured via performance during a socially adapted computerized laboratory risk-propensity task—the BART. In line with the predictions of the SRH of depressed mood (Allen & Badcock, 2003) and our hypotheses, we found that clinical depression is associated with a lower propensity for *social* risk-taking but not for individual risk-taking. This was indicated by a significant interaction effect on the BART that consisted of an average lower number of balloon pumps in the novel Social condition of the BART relative to never-depressed controls, with no such significant group-difference in the individual condition. Furthermore, within the Depressed group, the number of pumps in the Social condition was significantly lower than in the Individual condition, whereas this was not the case in the Control group.

Our findings are broadly in line with the predictions of the SRH (Allen & Badcock, 2003), which suggests a specific aversion for social risk-taking in depression, although it is important to note we did not directly measure such aversion here. The SRH posits that taking high social-risks is one of several potential mechanisms that could increase perceived social burden, thereby increasing the risk of exclusion or ostracism from the group (Allen & Badcock, 2003). Taking fewer risks with group resources would therefore be one way to help protect status and group inclusion. Previous research has rarely addressed social risk as a separable construct. However, our findings broadly align with a previous self-report questionnaire study on symptoms of depression, willingness to engage in risk-taking and subjective change in social status (Brewer & Olive, 2014). Higher levels of depressive symptomatology predicted a reduced willingness to engage in recreationally-risky behavior and a perceived fall in social status. The strength of the current study is that it tested this construct using a behavioral assessment of active risk-taking, as opposed to self-report measures of willingness to engage risk-taking, allowing us to control for self-presentation bias and the confounding effects of adoption of risky behaviors as coping strategies in depressed populations.

It is helpful to discuss potential alternative framings of the findings. One alternative account might be that clinical anxiety, and not clinical depression, was responsible for the observed results; in an evolutionary context, anxiety has been conceptualized as an adaptive response to threat (Bateson et al., 2011) and is associated with negative expectations for future outcomes (Shepperd et al., 2005). Accordingly, reduced risk taking has been characterized as a trait feature of anxiety (Giorgetta et al., 2012). In our sample, eight of the 25 Depressed participants had a past or current diagnosis of either Social Anxiety or Generalized Anxiety Disorder, and as would be expected scores on the BAI differed significantly between groups. Importantly, although we did not find any evidence of a moderating effect of anxiety symptoms measured via self-report questionnaire, future research could benefit from taking account of clinical anxiety disorder status between groups and/or including measures of momentary anxiety across the task. Moreover, investigating how evolutionary theories such as the SRH apply more broadly to comorbid depressive and anxiety disorders may have value in the development of transdiagnostic approaches to clinical practice (Dalgleish et al., 2020).

It is also important to disentangle social, as opposed to financial, risk when assessing social risk-taking with neuroeconomic paradigms. A social risk can be defined as any action that might lead to negative evaluation or exclusion by others, for example defending an unpopular opinion (Blakemore, 2018). Risky or irresponsible financial decisions might also lead to negative evaluations, with evidence suggesting that risk taking is affected by peer observation (L. Kessler et al., 2017) and the perceived risk values of one's peer group (Blakemore, 2018). In the current task, we conceptualized financial risk-taking using a group resource as a social risk; this rationale was based on the SRH’ assertion that an individual's risk of exclusion is determined by the ratio between their Social Value and Social Burden (Allen & Badcock, 2003), variables determined by the resources an individual is able to contribute to the group. Draining the group's financial resources would greatly increase an individual's Social Burden, and one's risk of exclusion. This method is consistent with previous studies which have measured social risk-taking in paradigms where financial decisions affect the payoffs of others (Bolton et al., 2015; Friedl et al., 2020).

In line with this, previous research suggests that framing of the BART as a “Loss” context, an opportunity to avoid losing money, may affect propensity for risk-taking (Gabriel & Williamson, 2010). However, prior literature suggests that a “Loss” framing may make participants either more (Benjamin & Robbins, 2007) or less risky (Gabriel & Williamson, 2010). In the current task, participants were informed that this was an opportunity to *win* tokens, a gains context, which has not been shown to have any significant associations with risky-behavior; however, the terminology in the instructions could have been expanded to include the potential for loss, thereby removing any directionality in the framing of the task.

Finally, two possible confounding factors in use of the BART with clinical participants are the role of executive function and delay discounting. There is evidence to suggest that executive function has a contribution to risk-taking behavior on the BART (Capone et al., 2016) and that working memory capacity specifically is associated with better performance and lower variability of performance on the BART in healthy adolescents (Blair et al., 2018). Depressed individuals have been found to have difficulties with working memory (Christopher & MacDonald, 2005) as well as other executive functions (Channon & Green, 1999). Depressed individuals have also been found to show a preference for short-term rewards (Pulcu et al., 2014). In the BART, the greater future payoff of a large balloon may thus be subject to more discounting by the depressed sample. It is possible that due to the flattening of reward-responsivity in depression (Allen et al., 2019) the prospect of a monetary reward was insufficient to motivate depressed participants to take risks which might obtain that greater future reward. Importantly, however, these limitations would apply equally to both the individual and social conditions of the task and would therefore struggle to explain the social specificity of the observed risk aversion.

This study does have some potential limitations. One possible limitation arises from the social manipulation prior to the BART whereby participants completed a PGG, which involved contributing to a group pot and provided the option to punish other group members. This task was chosen due to its emphasis on social responsibility and cooperation, however, it raises the possibility that participants’ risk preferences during the BART might have been influenced by group behavior observed during the PGG. However, importantly, we found no correlation between punishment or contributions on the PGG and pumps on the Social BART, and no difference in either punishment or contribution between clinical groups on the PGG (Supplemental Materials E) indicating that prior PGG parameters were unlikely to have influenced our findings. A second limitation is that participants completed the protocol in clinically homogeneous groups, which has possible confounding effects in that the experience of social interaction within a depressed group versus a never-depressed group may differ. Similarly, future work needs to investigate the motivations behind the social risk-aversion in clinical samples in order to provide more direct causal links between evolutionary theory and data; that is, are fewer pumps related to greater fear of exclusion/ostracism. Future replications could therefore involve mixed-diagnosis groups and recognize the inter-dependency of the data using a nested data structure accounting for group membership, prior social interaction and behavioral motivations. These putative limitations highlight the challenges in developing ecologically valid social protocols.

In conclusion, we found that clinical depression is associated with a lower propensity for social risk-taking, compared to the performance of healthy controls, and to non-social risk-taking. These findings offer preliminary support for the predictions of the SRH of depressed mood (Allen & Badcock, 2003) and have important implications for understanding the motivations of social behavior in depression.

## Supplementary Material

10.1037/abn0000797.supp

## Figures and Tables

**Table 1 tbl1:** Participant Demographics

Variable	Controls (*n* = 35)	Depressed (*n* = 27)	Between groups *t*-test and χ^2^	Total (*N* = 62)
Age			*t* = 0.82, *p* = .42	
Mean	43.22	46.79		44.77
Range	18–64	21–64		18–64
Gender (*n*, %)			χ^2^ = 1.43, *p* = .49	
Female	23 (66)	17 (63)		40 (64)
Male	12 (34)	10 (37)		22 (36)
Ethnicity			χ^2^ = 5.53, *p* = .24	
% Caucasian	82.9	88.9		85.5
% Chinese	8.4	—		4.8
% South Asian	5.8	—		3.2
% Mixed	2.9	11.1		6.5
UK education			χ^2^ = 3.17, *p* = .53	
% GCSE	17.1	14.8		16.1
% A level	28.6	18.5		24.2
% Bachelors	34.3	29.6		32.3
% Masters	14.3	18.5		16.1
% PhD	5.7	18.5		11.3
*Note*. GCSE = General Certificate of Secondary Education; A Level = Advanced Level.				

**Table 2 tbl2:** Past and Present Diagnostic Comorbidities in the Depressed Participants Assessed Using the Structured Clinical Interview for the DSM-5 in the Month Prior to Testing

Comorbidity	Past	Current	Total
Generalized anxiety disorder	1	4	5
Post-traumatic stress disorder	2	1	3
Social anxiety disorder	3	0	3
Panic disorder	1	2	3
Eating disorder	3	3	6

**Table 3 tbl3:** Scores on the Clinical and Social Status Measures for the Depressed and Control Groups, and Results of Independent Samples *t*-Tests Between Groups

Measure	Controls		Depressed		*t* statistic
	*M*	*SD*	*M*	*SD*	
1. BAI	4.79	6.03	14.60	7.36	5.57**
2. BDI-II	5.45	4.52	27.61	10.97	9.67**
3. ISQ	70.15	12.53	102.85	14.34	9.34**
4. SAIS:insecure	31.54	13.50	45.65	13.72	3.96**
5. SAIS:secure	34.18	6.89	23.11	12.11	4.16**
6. SBS	21.00	7.62	34.81	9.81	6.09**
**p < .01.					

## Footnotes

1 The assessment protocol involved a second rating of the Mood Module by CH if the diagnostic results differed from the original SCID (i.e., the patient was no longer in episode). However, there were no such divergences in the current sample.

2 Our a priori power calculation assumed a correlation of 0.6 across the two conditions of the BART. The correlation in the final analysis sample was 0.71, indicating that our power calculation was conservative.
