# Supplementary material for: Reduced Social Risk-Taking in Depression
Source: J Psychopathol Clin Sci. 2023 Feb;132(2):156–64. doi: 10.1037/abn0000797 (PMC9940639; doi:10.1037/abn0000797)
Supplement: Supplementary file 1 [file ABN-2021-2254_Suppl.docx]

**Supplemental Materials;**

**Reduced Social Risk-Taking in Depression**

Daisy Follett^1^, Caitlin Hitchcock^1^, Tim Dalgleish^1,2^*, Jason Stretton^1^*

*Joint senior authors

1. Medical Research Council Cognition and Brain Sciences Unit, University of Cambridge,

2. Cambridgeshire and Peterborough NHS Foundation Trust, Cambridge, UK

Corresponding author for this manuscript: jason.stretton@mrc-cbu.cam.ac.uk

Declarations of interest: none

This work was funded by the UK Medical Research Council (Grant Reference: SUAG/043 G101400)

**Supplementary Materials**

**Supplementary Materials A; Full Protocol**

Prior to the experimental session, participants completed four questionnaires at home; these were the Submissive Behaviour Scale (Allan & Gilbert, 1997), Striving to Avoid Inferiority Scale (Gilbert, Broomhead, Irons & McEwan, 2007), Interpersonal Sensitivity Measure (Boyce & Parker, 1989) and Involuntary Subordination Questionnaire (Sturman, 2011). The experimental session had five sections and was performed in groups of five participants at the Medical Research Council Cognition and Brain Sciences Unit in Cambridge, UK. The first section was completion of three questionnaires. This included two mood questionnaires, the Beck Depression Inventory II (Beck, Steer & Brown, 1996) and Beck Anxiety Inventory (Beck, Epstein, Brown & Steer, 1988), and one experimental questionnaire which formed part of the Actual-Ideal Task (reported elsewhere). This questionnaire had participants rate themselves from 1-9 on an inventory of 48 socially relevant attributes. The second section was a Public Goods Game, completed on laptops and programmed in z-Tree. In this neuroeconomic game participants decide individually how much of a resource (tokens) they wish to contribute to a group pot, which is multiplied and split equally. In our version, participants also had the option to punish other players by paying to deduct tokens from their total. This task is designed to investigate collaborative behaviour, and gave the participants an opportunity to work as a team and establish knowledge of the other players. The third section of the protocol involved two more questionnaires; the Hewitt-Flett Perfectionism Scale (Hewitt & Flett, 1990), and Narcissistic Personality Questionnaire (Raskin & Hall, 1979). The fourth section was the Balloon Analogue Risk Task, detailed here. The fifth section was completion of an experimental questionnaire which formed part of an Ultimatum Game Task. This widely-used neuroeconomic game has participants play in pairs comprising of one Offerer and one Receiver. Offerers decide how to split an amount of a resource between the pair. The Receiver can choose to accept the split, or to reject it, in which instance neither player receives anything. The questionnaire asked participants to imagine playing the game as an Offerer against each of their fellow players, and decide how much they might offer them given different amounts of money. Following this experimental session participants returned the next day for an imaging session which utilised their answers on the Actual-Ideal and Ultimatum Game questionnaires.

**Supplementary Materials B; Instructions for Participants.**

“In this game the goal is to maximise your winnings by collecting tokens, each worth 50p.

You will receive tokens for each balloon you pump up, according to its size. But if you pump it too far it will pop and you'll get nothing for that balloon.

Balloons differ in their maximum size - they can occasionally reach to almost the size of the screen but most will pop well before that.

Press SPACE to pump the balloon, or RETURN to bank the cash for this balloon and move onto the next. You will play this game twice; once for yourself, and once for your group. When playing for yourself, you keep all tokens and your total is private. When playing for the group, the tokens will be shared equally amongst the group, and your teammates will be informed of how much you won for them, and how often you popped the balloon. Which version you are playing will be clearly indicated on the next screen and throughout the game.”

**Supplementary Materials C: Pearson Correlations between Symptom and Social Status Measures and Corrected Mean Average number of Pumps on the Social and Individual BART**

To investigate whether the six individual difference measures might explain our observed results, Pearson correlations were conducted between these and the adjusted mean number of pumps per trial (Table S1). No significant correlations were observed either within or across groups with either condition.

|  |  | Controls | | Depressed | | Across Groups | |
| --- | --- | --- | --- | --- | --- | --- | --- |
| Variable |  | R Statistic | Sig. | R Statistic | Sig. | R statistic | Sig. |
| BAI | Social | -.30 | .08 | .15 | .47 | -.20 | .12 |
|  | Individual | -.31 | .07 | .20 | .31 | -.04 | .74 |
| BDI | Social | .03 | .84 | .16 | .43 | -.12 | .36 |
|  | Individual | .02 | .92 | .18 | .36 | .09 | .45 |
| IPSM | Social | -.14 | .43 | .02 | .92 | -.21 | .10 |
|  | Individual | -.08 | .65 | .24 | .23 | .06 | .65 |
| ISQ | Social | .16 | .37 | .16 | .42 | -.08 | .56 |
|  | Individual | .13 | .45 | .31 | .11 | .18 | .17 |
| SAIS:Insecure | Social | .04 | .80 | .01 | .95 | -.08 | .53 |
|  | Individual | -.18 | .30 | .22 | .26 | .00 | .99 |
| SAIS:Secure | Social | .05 | .77 | -.01 | .98 | .14 | .27 |
|  | Individual | .22 | .20 | -.12 | .56 | .02 | .86 |
| SBS | Social | -.10 | .57 | .00 | .99 | -.19 | .13 |
|  | Individual | -.14 | .41 | .07 | .72 | -.01 | .93 |
| **Table S1.** No significant correlations were found between the symptom and social status measures and corrected average number of pumps on either the Social or Individual conditions of the BART | | | | | | | |

Moreover, a repeated measures ANCOVA was run with all individual difference measures included as covariates. This showed no significant effects of any covariates and the adjusted mean number of pumps in either the individual or social conditions and the interaction reported between Condition and Group remained significant, (F(1,49)=9.79, p=0.03, η_p_2 =.17).

**Supplementary Materials D; Results of Repeated Measures ANCOVA including BAI score as a covariate**

A repeated measures ANCOVA was conducted with BAI as a covariate (Table S2). This found no significant interaction between BAI score and the difference in number of pumps between the two conditions, *F*(1,60)=0.05, *p*=0.82, ηp^2^ =.00, and the interaction reported between Condition and Group remained significant, *F*(1,60)=6.77, *p*=0.012, ηp^2^ =.11.

|  | df | Mean Square | F | Sig. | Partial ETA Squared |
| --- | --- | --- | --- | --- | --- |
| Condition x BAI | 1 | 1.29 | .05 | .82 | .001 |
| Condition x Group | 1 | 169.03 | 6.77 | .01 | .105 |
| **Table S2.** No interaction was found between BAI score and difference in the Number of Pumps between the two Conditions | | | | | |

**Supplementary Materials E; Performance on the prior Public Goods Game**

To investigate whether performance on the prior Public Goods Game influenced the observed differences between our samples, Independent samples t-test were run between MDD and Control groups on each of the five key variables of the PGG; Final Total Tokens, Mean Percentage Contribution, Mean Raw Contribution, Mean Percentage Punishpoints and Mean Raw Punishpoints (where “percentage” variables represented the percentage of the participants tokens contributed or utilised in punishing others). No significant differences were observed between groups; these results are presented in Table S3.

|  | Controls | | Depressed | |  |  |
| --- | --- | --- | --- | --- | --- | --- |
| Variable | M | SD | M | SD | T statistic | Sig. |
| Final Total | 41.58 | 17.69 | 41.36 | 26.16 | .035 | .972 |
| Mean Percentage Contribution | .39 | .18 | .41 | .19 | -.333 | .741 |
| Mean Raw Contribution | 10.45 | 3.32 | 9.81 | 4.13 | .599 | .552 |
| Mean Percentage Punishpoints | .03 | .04 | .06 | .11 | -1.034 | .306 |
| Mean Raw Punishpoints | .91 | .91 | .91 | 1.32 | -.012 | .990 |
| **Table S3.** Independent samples t-test found no significant differences between groups on the 5 key variables | | | | | | |

To investigate this on the individual level, a Pearson correlation analysis was run between the corrected number of pumps on the Social BART and two key variables in the PGG; the Final Total Tokens accrued and the Mean number of Punishpoints allocated by the participants group during the PGG. No significant correlations were observed either when the samples were analysed separately or combined (Table S4).

|  | Final Total | | Punishpoints | |
| --- | --- | --- | --- | --- |
| Group | R | Sig. | R | Sig. |
| Across Groups | -.102 | .431 | -.033 | .797 |
| Controls | .056 | .748 | -.99 | .572 |
| Depressed | -.225 | .260 | .173 | .389 |
| **Table S4.**No correlations were observed between the number of pumps an individual made in the Social Condition of BART and either the Total Tokens accrued or the number of Punishpoints allocated by their group during the prior PGG. | | | | |
